# Supplementary material for: Lizards, Lineage and Latitude: Behavioural Responses to Microclimate Vary Latitudinally and Show Limited Acclimatisation to a Common Environment After Two Years
Source: Biology (Basel). 2025 May 28;14(6):622. doi: 10.3390/biology14060622 (PMC12189329; doi:10.3390/biology14060622)
Supplement: Supplementary file 1 [file biology-14-00622-s001.zip › biology-3558373-supplementary.pdf]

Supplementary S1

S1.1: Translocation

Site preparation

Ten enclosures were built in 2019, using 300 mm high lengths of sheet metal buried approximately 100 mm in the ground and riveted together to form a 2 × 5 grid of 25 × 25 m enclosures. Timber artificial burrows made from 30 × 300 mm hardwood dowel were installed in accordance with Milne (2000), Souter *et al.* (2007) and Pettigrew & Bull (2011) (Figure S1.1.1). Each enclosure was divided into 5 × 5 m sections, and an orange marker peg installed at each linear intersection. A cluster of 3 × 400 mm deep holes were drilled on the South-west side of each orange marker peg using a 30 × 400 mm drill bit and cordless drill. These holes were spaced 200 mm apart (centre to centre) in a triangular formation. A timber artificial burrow was tapped into each hole with a hammer until the top of the burrow was flush with the ground (Figure S1.1.1).

(a)

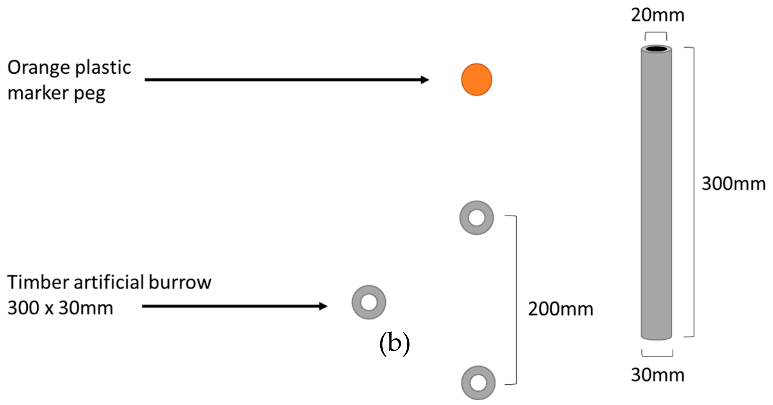

Figure S1.1.1 (a) Layout of artificial burrow clusters at the translocation site at Tarlee, (b) dimensions of an individual burrow

Capture and translocation of pygmy bluetongue lizards 2020

Pygmy bluetongues were located during August through September 2020 at Kapunda and Burra, and in January 2021 in Jamestown, Burra and Kapunda South Australia by searching sites with previously recorded populations. Transects were walked and appropriately sized burrows were marked with a wire and plastic flag. Marked burrows were then inspected with an Inline portable endoscope (Yatek M615FM) and burrows with pygmy bluetongue lizard occupants marked with flagging tape. Semi-permanent orange plastic pegs were installed 200 mm from the burrow entrance at pygmy bluetongue lizard occupied burrows and a GPS waypoint taken using a Garmin e-trek. Approximately 30 lizards were marked at each site. Lizards were caught using the fishing rod and mealworm lure technique as per Milne (2000). Between September 2020 and March 2021, 32 adult lizards were caught at each site and transported in cloth catch bags inside lidded boxes, one bag and box per individual and transported to Flinders University Animal House where they were quarantined prior to release. Lizards were given a unique toe-clip ID (Figure S1.1.2) and SVL (snout to vent length) (mm) and mass (g) taken on arrival and on

 https://doi.org/10.3390/biology14060622

departure from the animal house. This project was carried out in accordance with State Government permit G25011, ethics approval E453-17 and Department of Environment and Water 'Take from the wild' permit 20210331.

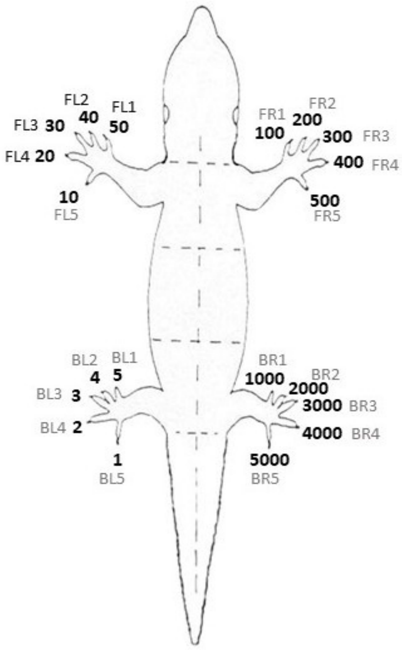

Figure S1.1.2 Schematic for toe-clip numbers used to identify individual lizards

Release at Tarlee

On 21st October 2020 and 16th April 2021 pygmy bluetongue lizards were released into the Tarlee translocation enclosures. One lizard was released per cluster of three artificial burrows with a soft release enclosure around the cluster (Figure S1.1.3). Lineages were released into separate enclosures, Jamestown lizards into enclosures 3 and 4, Burra lineage lizards into enclosures 5 and 6, and Kapunda lizards into enclosures 7 and 10 (Figure S1.1.4). Each lizard was fed two mealworms on the day of release. Three days after release the soft release enclosures were removed and lizards were fed two mealworms as per (Ebrahimi & Bull 2012, 2013). Lizards were left to settle for two weeks before behavioural monitoring began.

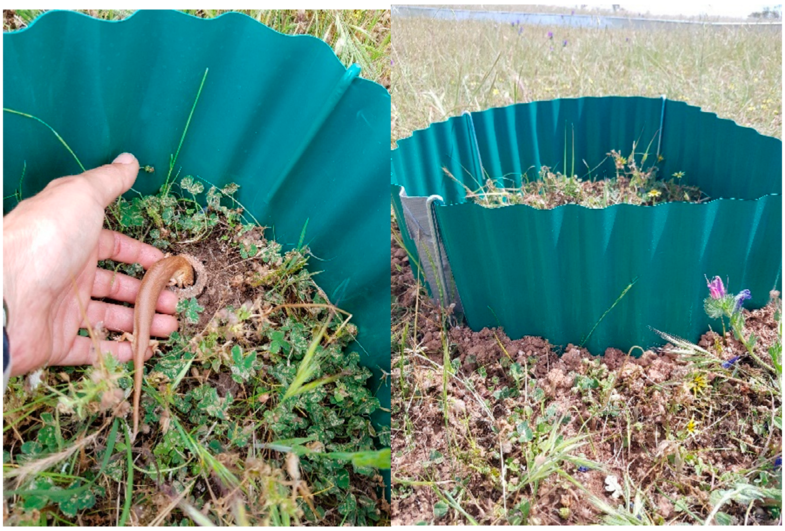

Figure S1.1.3 Soft release enclosures at the translocation site at Tarlee.

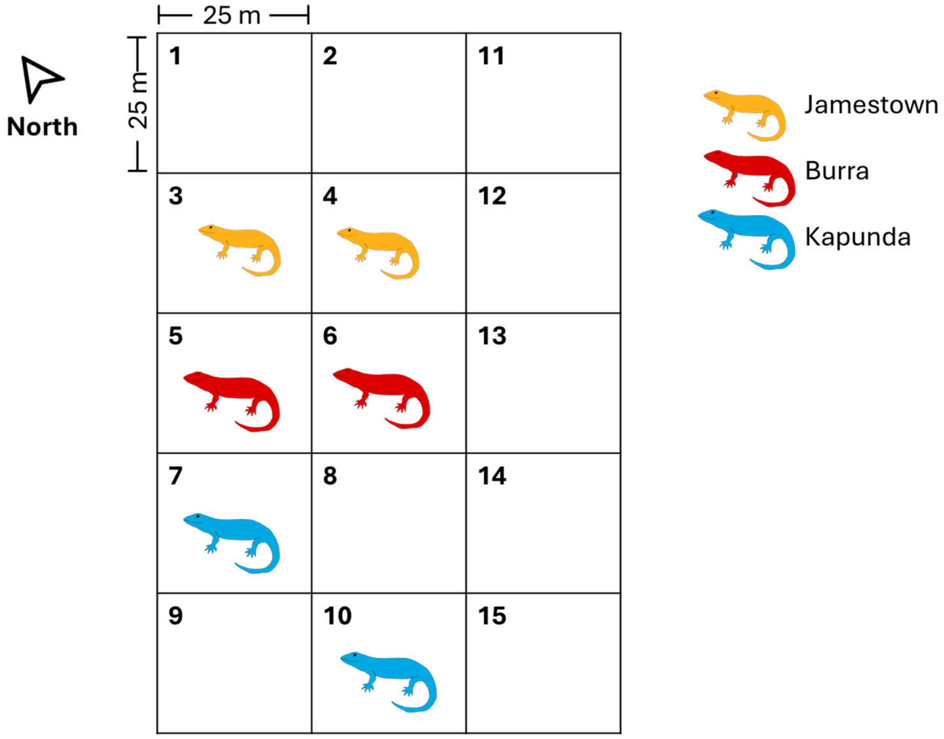

Figure S1.1.4 Overview of translocation enclosures at Tarlee showing the location of the Jamestown, Kapunda and Burra lineage translocated individuals

S1.2: Behaviour

Table S1.2.1 Ethogram of behaviours scored in the approach distance assay of pygmy bluetongue (*Tiliqua adelaidensis*) lizards from four sites, northern, mid, southern and translocation, and definitions.

Behaviour	Description
Basking	Lizard can be seen in their burrow: 'Head Only' or 'Head and Forelimbs' (Milne et al. 2003). Head forelimbs and torso was also scored.
Emerge	Head, forelimbs and hind limbs out (Milne et al. 2003).
Other	Any notable behaviour not listed above.

S1.3: Environmental data collection

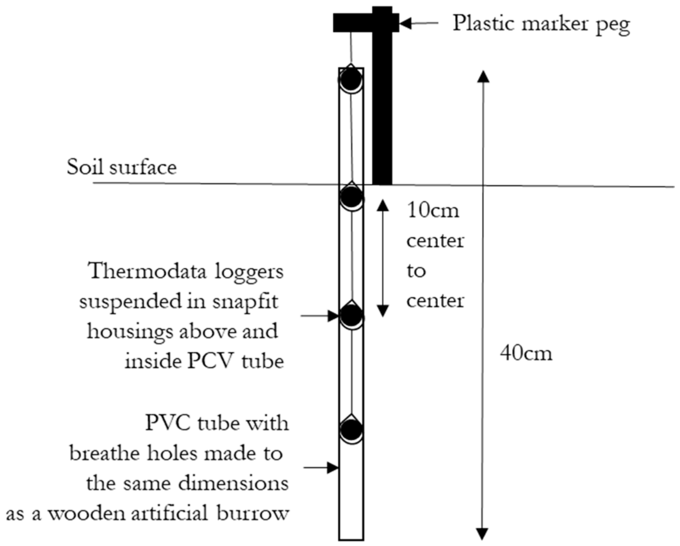

Figure S1.3.1 positions of ibutton data loggers (maxim integrated n.d.) inside an artificial burrow made from PVC tube 250 mm diameter and perforated to allow transfer of heat and moisture from the substrate to the dataloggers as per a natural or timber burrow. Four loggers were deployed per artificial burrow, at the base, the middle, the top, and above ground for ambient temperature, three replicates per site for all sites.

Supplementary S2

Table S2.1: Summary statistics of pygmy bluetongue (*Tiliqua adelaidensis*) approach distance behavioural assay from the 2022 to 2023 active season.

Variable	N	Mean	Std. Dev.	Min	Pctl. 25	Pctl. 75	Max
vs_sites	3133						
... Burra	581	19%					
... Jamestown	584	19%					
... Kapunda	179	6%					
... Tarlee	1789	57%					
vs_lineages	3133						
... Burra	1164	37%					
... Jamestown	1221	39%					
... Kapunda	748	24%					
vn_retreat_metres	705	2	1.1	0.19	1.2	2.5	8
vl_actives	3133						
... No	2428	77%					
... Yes	705	23%					
vl_translocated	3133						
... No	1344	43%					
... Yes	1789	57%					
tstat	3133						
... Wild	1344	43%					
... Translocated	1789	57%					
lineage	3133						
... Mid	1164	37%					
... Northern	1221	39%					
... Southern	748	24%					
vs_burrows_f	3116						

Table S2.2 PCA axes and relative importance for environmental variables taken at all sites (Jamestown, Kapunda, Burra, Tarlee) over the October to December 2022 activity season

```
                         PC1    PC2    PC3
Standard deviation     2.2392 1.0488 1.0281
Proportion of Variance 0.6267 0.1375 0.1321
Cumulative Proportion  0.6267 0.7642 0.8963
```

Table S2.3 Results of generalized additive mixed model of wild pygmy bluetongue lizard (*Tiliqua adelaidensis*) approach distance (log m) by lineage (northern, mid and southern), and PC1, PC2 and PC3

```
Parametric coefficients:
                Estimate Std. Error t value Pr(>|t|)
(Intercept)       0.6932     0.3254   2.130   0.0346 *
lineageMid        0.1689     0.3891   0.434   0.6649
lineageSouthern   1.3176     2.7312   0.482   0.6301
---
```

```
Signif. codes:  0 ‘***’ 0.001 ‘**’ 0.01 ‘*’ 0.05 ‘.’ 0.1 ‘ ’ 1

Approximate significance of smooth terms:
                          edf Ref.df     F p-value
s(PC1):lineageNorthern  4.769  5.672 2.792 0.01770 *
s(PC1):lineageMid       3.673  4.334 0.791 0.57854
s(PC1):lineageSouthern  1.723  2.056 0.210 0.82220
s(PC2):lineageNorthern  3.663  4.253 3.306 0.01005 *
s(PC2):lineageMid       2.842  3.221 1.066 0.36722
s(PC2):lineageSouthern  2.356  2.823 1.394 0.34236
s(PC3):lineageNorthern  2.842  3.311 4.051 0.01361 *
s(PC3):lineageMid       3.847  4.375 0.971 0.33758
s(PC3):lineageSouthern  1.835  2.106 0.246 0.83131
s(vs_burrows_f)        13.064 30.000 0.879 0.00407 **
---
Signif. codes:  0 ‘***’ 0.001 ‘**’ 0.01 ‘*’ 0.05 ‘.’ 0.1 ‘ ’ 1

R-sq.(adj) =  0.295   Deviance explained = 43.8%
-REML = 168.72  Scale est. = 0.19867   n = 210
```

Table S2.4 Results of generalized additive mixed model of translocated pygmy bluetongue lizard (*Tiliqua adelaidensis*) approach distance (log m) by lineage (northern, mid and southern), and PC1, PC2 and PC3

```
Parametric coefficients:
                 Estimate Std. Error t value Pr(>|t|)
(Intercept)        0.7772     0.1791   4.339 2.43e-05 ***
lineageMid        -0.0387     0.2035  -0.190    0.849
lineageSouthern   -0.2075     0.1949  -1.065    0.288
---
Signif. codes:  0 ‘***’ 0.001 ‘**’ 0.01 ‘*’ 0.05 ‘.’ 0.1 ‘ ’ 1

Approximate significance of smooth terms:
                         edf Ref.df     F p-value
s(PC1):lineageNorthern 1.000  1.000 0.003  0.9570
s(PC1):lineageMid      1.614  1.887 0.473  0.5439
s(PC1):lineageSouthern 1.000  1.000 0.112  0.7385
s(PC2):lineageNorthern 2.537  3.112 1.107  0.3241
s(PC2):lineageMid      1.000  1.000 0.136  0.7127
s(PC2):lineageSouthern 1.000  1.000 1.354  0.2462
s(PC3):lineageNorthern 4.252  4.973 2.858  0.0241 *
s(PC3):lineageMid      3.176  3.579 1.196  0.2697
s(PC3):lineageSouthern 5.713  6.685 0.538  0.7969
```

```
s(vs_burrows_f)       6.423 74.000 0.110  0.1388
---
Signif. codes:  0 ‘***’ 0.001 ‘**’ 0.01 ‘*’ 0.05 ‘.’ 0.1 ‘ ’ 1

R-sq.(adj) =  0.169   Deviance explained = 29.1%
GCV = 0.31687  Scale est. = 0.26893   n = 203
```

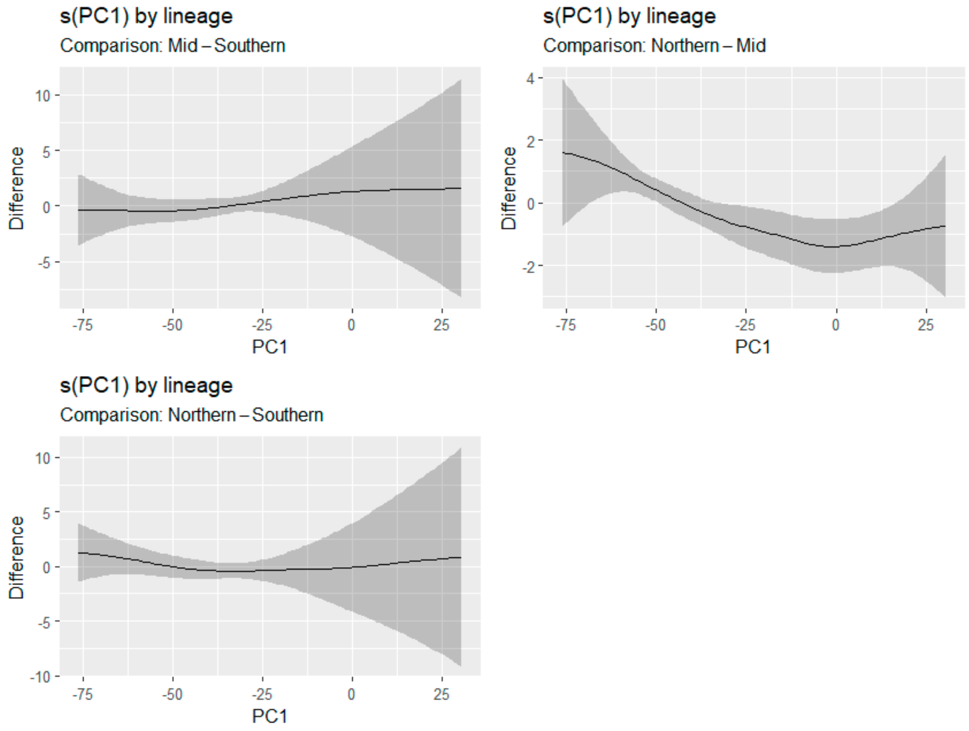

Figure S2.1: Pairwise comparisons of differences between smooth curves for PC1 by lineage for wild lizards in the north, mid and south of the current pygmy bluetongue (*Tiliqua adelaidensis*) range. Curve sections with significant difference do not overlap with zero.

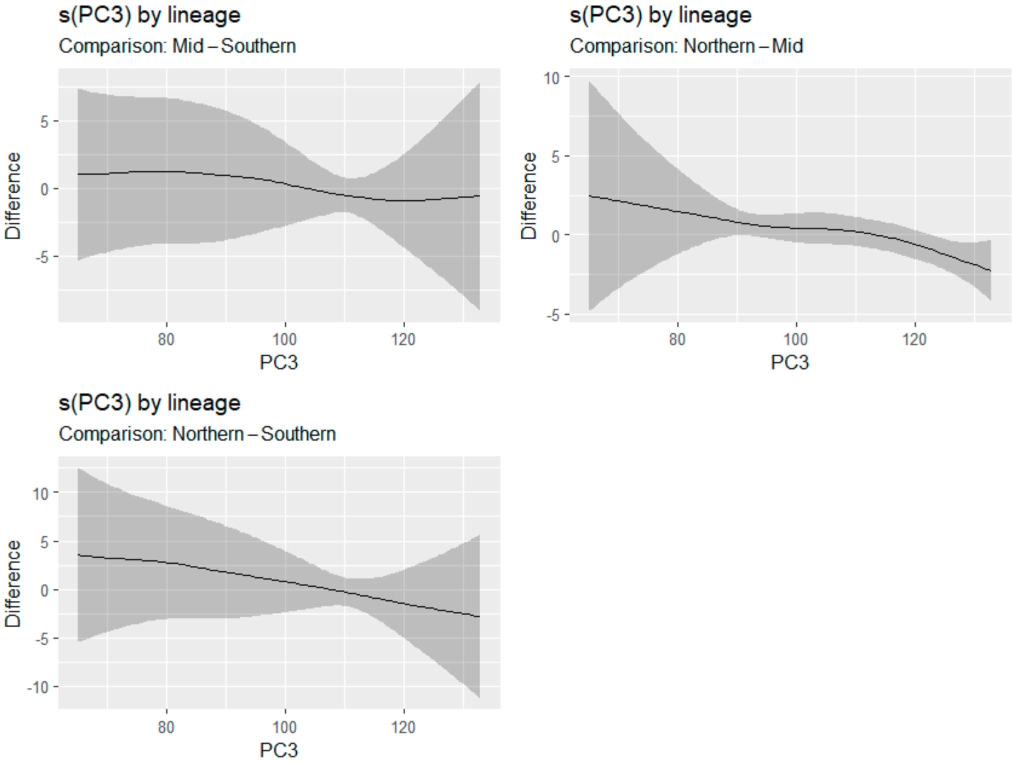

Figure S2.2: Pairwise comparisons of differences between smooth curves for PC3 by lineage for wild lizards in the north, mid and south of the current pygmy bluetongue (*Tiliqua adelaidensis*) range. Curve sections with significant difference do not overlap with zero.

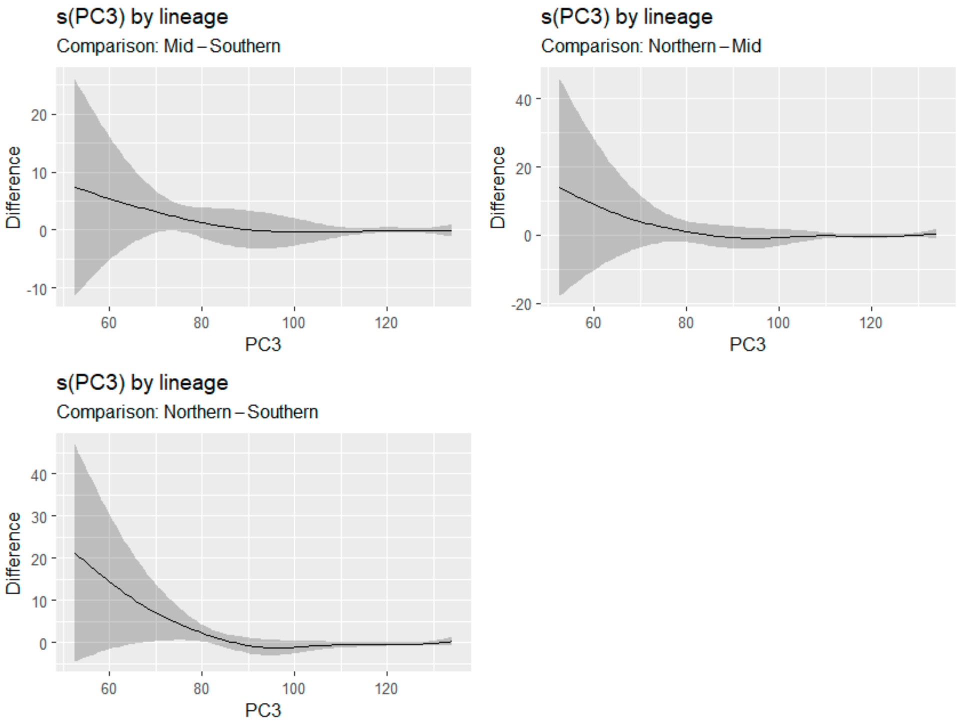

Figure S2.3: Pairwise comparisons of differences between smooth curves for PC3 by lineage for translocated lizards in the north, mid and south of the current pygmy bluetongue (*Tiliqua adelaidensis*) range. Curve sections with significant difference do not overlap with zero.

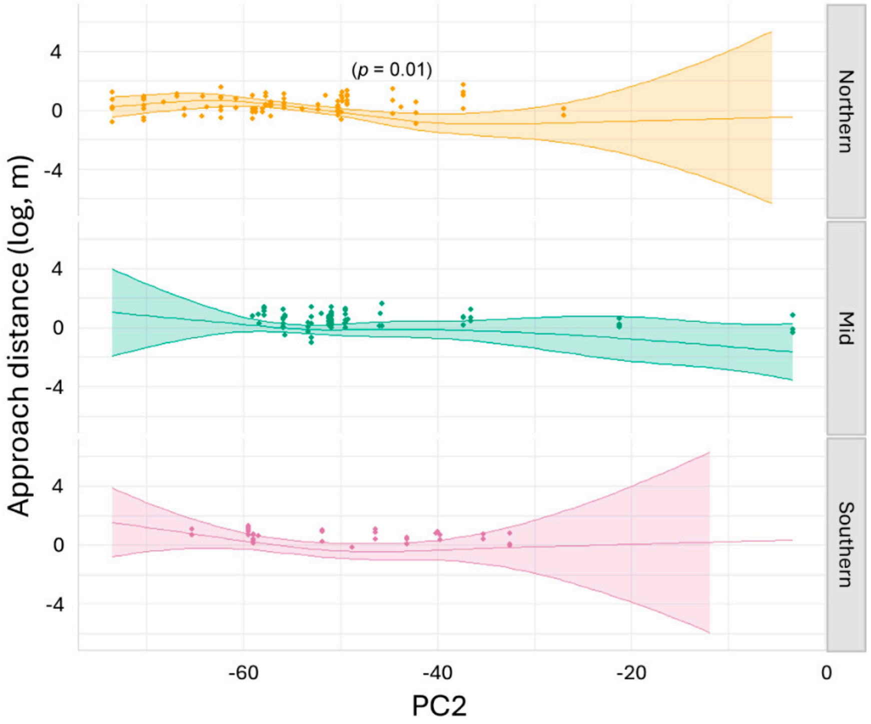

Figure S2.4: Model predicted approach distance (log, m) and raw data points for northern (Jamestown), mid (Burra), and southern (Kapunda) lineages of pygmy bluetongue lizard (*Tiliqua adelaidensis*) in the wild for PC2 (decreasing ambient temperature and increasing mid and base of burrow temperature and ambient relative humidity as PC2 values increase) and lineage. Lines depict model predictions, coloured bands depict 95% C.I and points depict the raw data.

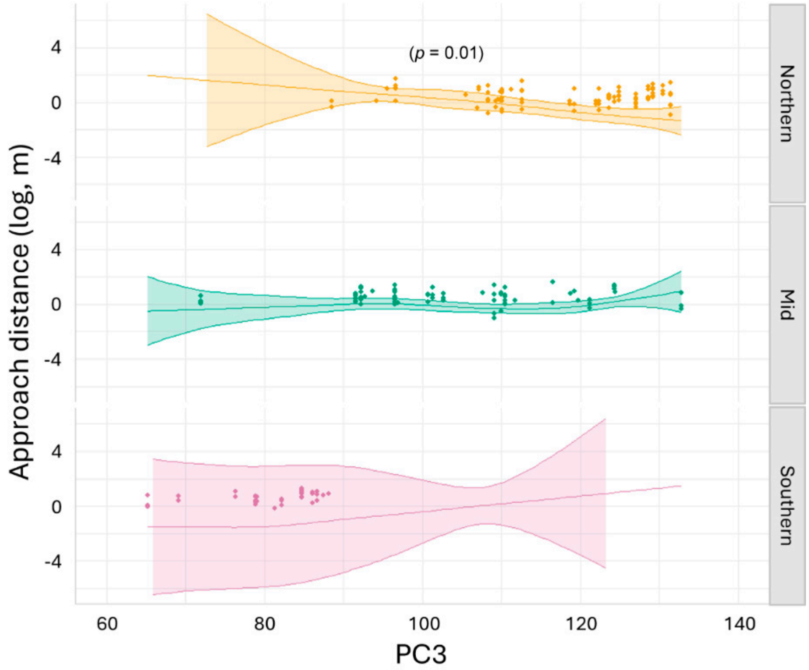

Figure S2.5: Model predicted approach distance (log, m) and raw data points for northern (Jamestown), mid (Burra), and southern (Kapunda) lineages of pygmy bluetongue lizard (*Tiliqua adelaidensis*) in the wild for PC3 (increasing burrow relative humidity as PC3 values increase) and

lineage. Lines depict model predictions, coloured bands depict 95% C.I and points depict the raw data.

Table S2.5: Number of unique burrows observed at each site for each lineage of pygmy bluetongue (Tiliqua adelaidensis) lizards.

Site	Lineage	Number of unique burrows observed
Burra	Burra	68
Jamestown	Jamestown	68
Kapunda	Kapunda	40
Tarlee	Burra	107
Tarlee	Jamestown	115
Tarlee	Kapunda	101

Supplementary S3

Table S3.1: Bureau of meteorology rainfall records (mm) for the months of October, November and December 2022 for the four sites, Jamestown (northern), Burra (mid-range), Kapunda (southern) and Tarlee (translocation).

**Site**	**Oct-22**	**Nov-22**	**Dec-22**	**Total (mm)**
Tarlee	122.2	115.4	16.2	253.8
Kapunda	144.3	135.2	16.8	296.3
Burra	140	137.6	20	297.6
Jamestown	93.4	131.8	6	231.2

Table S3.2: Temperature (◦C) and relative humidity (%) 2022 to 2023 season means and standard deviations for the four sites; Jamestown (northern), Burra (mid-range), Kapunda (southern) and Tarlee (translocation), in the base of the burrow (base) and 10 mm above the surface (ambient).

**Position**	**Site**	**Temperature (◦C)**	**SD**	**Humidity (%)**	**SD**
Base	Tarlee	22.96	5.26	89.53	20.0
	Kapunda	18.79	2.48	59.04	4.40
	Burra	21.59	4.93	89.19	13.0
	Jamestown	20.9	4.08	94.97	11.14

Ambient	Tarlee	22.56	12.27	64.09	31.25
	Kapunda	18.44	11.36	79.25	32.3
	Burra	19.92	10.37	61.27	32.68
	Jamestown	21.6	14.0	68.62	35.97

Table S3.3: Mean rainfall (mm) calculated from Bureau of Meteorology rainfall data for 2011 to 2025 for the months of October, November and December for the four sites; Jamestown (northern), Burra (mid-range), Kapunda (southern) and Tarlee (translocation). Spring quarter is calculated from the mean rainfall (mm) of October, November and December 2011 to 2025.

	October	November	December	Spring quarter	Annual
Burra	35.90	42.40	23.30	101.60	376.80
Jamestown	37.19	48.06	31.07	116.32	493.76
Kapunda	38.29	38.84	21.15	98.27	458.79
Tarlee	30.00	33.43	22.63	86.06	445.79

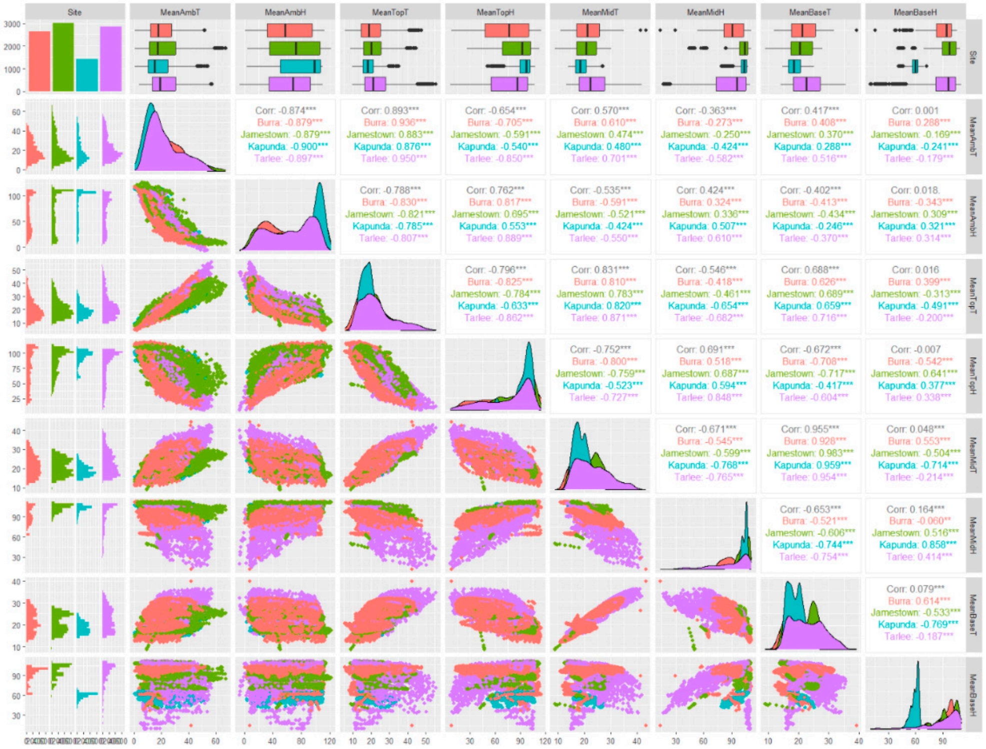

Figure S3.1: Correlation matrix for data logger positions inside burrows separated by sites using seasonal means, where MeanTopT = Mean top temperature, MeanTopH = Mean top humidity, MeanBaseT = Mean base temperature, MeanBaseH = Mean base humidity, for the four sites: Jamestown (northern), Burra (mid-range), Kapunda (southern) and Tarlee (translocation).

References

1. Parmesan, C. Ecological and Evolutionary Responses to Recent Climate Change. *Annu. Rev. Ecol. Evol. Syst.* **2006**, *37*, 637–669. https://doi.org/10.1146/annurev.ecolsys.37.091305.110100.
2. Woods, H.A.; Dillon, M.E.; Pincebourde, S. The Roles of Microclimatic Diversity and of Behavior in Mediating the Responses of Ectotherms to Climate Change. *J. Therm. Biol.* **2015**, *54*, 86–97. https://doi.org/10.1016/j.jtherbio.2014.10.002.
3. Sinclair, B.J.; Marshall, K.E.; Sewell, M.A.; Levesque, D.L.; Willett, C.S.; Slotsbo, S.; Dong, Y.; Harley, C.D.G.; Marshall, D.J.; Helmuth, B.S.; et al. Can We Predict Ectotherm Responses to Climate Change Using Thermal Performance Curves and Body Temperatures? *Ecol. Lett.* **2016**, *19*, 1372–1385. https://doi.org/10.1111/ele.12686.
4. Clusella-Trullas, S.; Blackburn, T.M.; Chown, S.L. Climatic Predictors of Temperature Performance Curve Parameters in Ectotherms Imply Complex Responses to Climate Change. *Am. Nat.* **2011**, *177*, 738–751. https://doi.org/10.1086/660021.
5. Kefford, B.J.; Ghalambor, C.K.; Dewenter, B.; Poff, N.L.; Hughes, J.; Reich, J.; Thompson, R. Acute, Diel, and Annual Temperature Variability and the Thermal Biology of Ectotherms. *Glob. Change Biol.* **2022**, *28*, 6872–6888. https://doi.org/10.1111/gcb.16453.
6. Pintor, A.F.V.; Schwarzkopf, L.; Krockenberger, A.K. Hydroregulation in a Tropical Dry-Skinned Ectotherm. *Oecologia* **2016**, *182*, 925–931.
7. Smith, A.L.; Gardner, M.G.; Fenner, A.L.; Bull, C.M. Restricted Gene Flow in the Endangered Pygmy Bluetongue Lizard (*Tiliqua Adelaidensis*) in a Fragmented Agricultural Landscape. *Wildl. Res.* **2009**, *36*, 466. https://doi.org/10.1071/WR08171.
8. Miller, K.A.; Miller, H.C.; Moore, J.A.; Mitchell, N.J.; Cree, A.; Allendorf, F.W.; Sarre, S.D.; Keall, S.N.; Nelson, N.J. Securing the Demographic and Genetic Future of Tuatara through Assisted Colonization: Assisted Colonization of Tuatara. *Conserv. Biol.* **2012**, *26*, 790–798. https://doi.org/10.1111/j.1523-1739.2012.01902.x.

9. Böhm, M.; Collen, B.; Baillie, J.E.M.; Bowles, P.; Chanson, J.; Cox, N.; Hammerson, G.; Hoffmann, M.; Livingstone, S.R.; Ram, M.; et al. The Conservation Status of the World's Reptiles. *Biol. Conserv.* **2013**, *157*, 372–385. https://doi.org/10.1016/j.biocon.2012.07.015.
10. Clusella-Trullas, S.; Chown, S.L. Lizard Thermal Trait Variation at Multiple Scales: A Review. *J. Comp. Physiol. B* **2014**, *184*, 5–21. https://doi.org/10.1007/s00360-013-0776-x.
11. Delean, S.; Bull, C.M.; Brook, B.W.; Heard, L.M.B.; Fordham, D.A. Using Plant Distributions to Predict the Current and Future Range of a Rare Lizard. *Divers. Distrib.* **2013**, *19*, 1125–1137. https://doi.org/10.1111/ddi.12050.
12. Urban, M.C.; Richardson, J.L.; Freidenfelds, N.A. Plasticity and Genetic Adaptation Mediate Amphibian and Reptile Responses to Climate Change. *Evol. Appl.* **2014**, *7*, 88–103. https://doi.org/10.1111/eva.12114.
13. Chapple, D.G.; Roll, U.; Böhm, M.; Aguilar, R.; Amey, A.P.; Austin, C.C.; Baling, M.; Barley, A.J.; Bates, M.F.; Bauer, A.M.; et al. Conservation Status of the World's Skinks (Scincidae): Taxonomic and Geographic Patterns in Extinction Risk. *Biol. Conserv.* **2021**, *257*, 109101. https://doi.org/10.1016/j.biocon.2021.109101.
14. Sih, A.; Bell, A.; Johnson, J.C. Behavioral Syndromes: An Ecological and Evolutionary Overview. *Trends Ecol. Evol.* **2004**, *19*, 372–378. https://doi.org/10.1016/j.tree.2004.04.009.
15. Ribeiro, Â.M.; Lloyd, P.; Bowie, R.C.K. A Tight Balance between Natural Selection and Gene Flow in a Southern African Arid-Zone Endemic Bird: Spatial Heterogeneous Environments and Gene Flow. *Evolution* **2011**, *65*, 3499–3514. https://doi.org/10.1111/j.1558-5646.2011.01397.x.
16. Pavlova, A.; Amos, J.N.; Joseph, L.; Loynes, K.; Austin, J.J.; Keogh, J.S.; Stone, G.N.; Nicholls, J.A.; Sunnucks, P. Perched at the Mito-Nuclear Crossroads: Divergent Mitochondrial Lineages Correlate with Environment in the Face of Ongoing Nuclear Gene Flow in an Australian Bird. *Evolution* **2013**, *67*, 3412–3428. https://doi.org/10.1111/evo.12107.
17. Campbell-Staton; Bare, A.; Losos, J.B.; Edwards, S.V.; Cheviron, Z.A. Physiological and Regulatory Underpinnings of Geographic Variation in Reptilian Cold Tolerance across a Latitudinal Cline. *Mol. Ecol.* **2018**, *27*, 2243–2255. https://doi.org/10.1111/mec.14580.
18. Trewartha, D.M.; Clayton, J.L.; Godfrey, S.S.; Gardner, M.G. Heat Water and Reptiles—Do the Hydro-thermal Properties of Animals at the Source Location Persist at the Translocation Site? *Anim. Conserv.* **2024**, *28*, 33–48. https://doi.org/10.1111/acv.12942.
19. Abram, P.K.; Boivin, G.; Moiroux, J.; Brodeur, J. Behavioural Effects of Temperature on Ectothermic Animals: Unifying Thermal Physiology and Behavioural Plasticity: Effects of Temperature on Animal Behaviour. *Biol. Rev.* **2017**, *92*, 1859–1876. https://doi.org/10.1111/brv.12312.
20. Neilson, K.A. Evaporative Water Loss as a Restriction on Habitat Use in Endangered New Zealand Endemic Skinks. *J. Herpetol.* **2002**, *36*, 342–348.
21. Cowles, R.B.; Bogert, C.M. A Preliminary Study of the Thermal Requirements of Desert Reptiles. *Q. Rev. Biol.* **1945**, *20*, 170. https://doi.org/10.1086/394795.
22. Stevenson, R.D. The Relative Importance of Behavioral and Physiological Adjustments Controlling Body Temperature in Terrestrial Ectotherms. *Am. Nat.* **1985**, *126*, 362–386. https://doi.org/10.1086/284423.
23. Kearney, M.; Porter, W. Mechanistic Niche Modelling: Combining Physiological and Spatial Data to Predict Species' Ranges. *Ecol. Lett.* **2009**, *12*, 334–350. https://doi.org/10.1111/j.1461-0248.2008.01277.x.
24. Leal, M.; Gunderson, A.R. Rapid Change in the Thermal Tolerance of a Tropical Lizard. *Am. Nat.* **2012**, *180*, 815–822. https://doi.org/10.1086/668077.
25. Sinervo, B.; Lara Reséndiz, R.A.; Miles, D.B.; Lovich, J.E.; Rosen, P.C.; Gadsden, H.; Gaytán, G.C.; Tessaro, P.G.; Luja, V.H.; Huey, R.B.; et al. Climate Change and Collapsing Thermal Niches of Desert Reptiles and Amphibians: Assisted Migration and Acclimation Rescue from Extirpation. *Sci. Total Environ.* **2024**, *908*, 168431. https://doi.org/10.1016/j.scitotenv.2023.168431.
26. Wild, K.H.; Huey, R.B.; Pianka, E.R.; Clusella-Trullas, S.; Gilbert, A.L.; Miles, D.B.; Kearney, M.R. Climate Change and the Cost-of-Living Squeeze in Desert Lizards. *Science* **2025**, *387*, 303–309. https://doi.org/10.1126/science.adq4372.
27. Kingsolver, J.G.; Diamond, S.E.; Buckley, L.B. Heat Stress and the Fitness Consequences of Climate Change for Terrestrial Ectotherms. *Funct. Ecol.* **2013**, *27*, 1415–1423. https://doi.org/10.1111/1365-2435.12145.
28. Daltry, J.C.; Ross, T.; Thorpe, R.S.; Wuster, W. Evidence That Humidity Influences Snake Activity Patterns: A Field Study of the Malayan Pit Viper *Calloselasma rhodostoma*. *Ecography* **1998**, *21*, 25–34. https://doi.org/10.1111/j.1600-0587.1998.tb00391.x.
29. Kearney, M.; Munns, S.L.; Moore, D.; Malishev, M.; Bull, C.M. Field Tests of a General Ectotherm Niche Model Show How Water Can Limit Lizard Activity and Distribution. *Ecol. Monogr.* **2018**, *88*, 672–693. https://doi.org/10.1002/ecm.1326.

30. Le Galliard, J.-F.; Rozen-Rechels, D.; Lecomte, A.; Demay, C.; Dupoué, A.; Meylan, S. Short-Term Changes in Air Humidity and Water Availability Weakly Constrain Thermoregulation in a Dry-Skinned Ectotherm. *PLoS ONE* **2021**, *16*, e0247514. https://doi.org/10.1371/journal.pone.0247514.
31. Stamps, J.A. Rainfall, Activity and Social Behaviour in the Lizard, *Anolis aeneus*. *Anim. Behav.* **1976**, *24*, 603–608. https://doi.org/10.1016/S0003-3472(76)80074-7.
32. Crowley, S.R. The Effect of Desiccation upon the Preferred Body Temperature and Activity Level of the Lizard Sceloporus Undulatus. *Copeia* **1987**, *1987*, 25. https://doi.org/10.2307/1446033.
33. Jones, S.M.; Waldschmidt, S.R.; Potvin, M.A. An Experimental Manipulation of Food and Water: Growth and Time-Space Utilization of Hatchling Lizards (*Sceloporus undulatus*). *Oecologia* **1987**, *73*, 53–59. https://doi.org/10.1007/BF00376977.
34. Lorenzon, P.; Clobert, J.; Oppliger, A.; John-Alder, H. Effect of Water Constraint on Growth Rate, Activity and Body Temperature of Yearling Common Lizard (*Lacerta vivipara*). *Oecologia* **1999**, *118*, 423–430. https://doi.org/10.1007/s004420050744.
35. Kerr, G.D.; Bull, C.M. Field Observations of Extended Locomotor Activity at Sub-Optimal Body Temperatures in a Diurnal Heliothermic Lizard (*Tiliqua Rugosa*). *J. Zoology* **2004**, *264*, 179–188. https://doi.org/10.1017/S0952836904005734.
36. Sannolo, M.; Carretero, M.A. Dehydration Constrains Thermoregulation and Space Use in Lizards. *PLoS ONE* **2019**, *14*, e0220384. https://doi.org/10.1371/journal.pone.0220384.
37. Rozen-Rechels, D.; Rutschmann, A.; DupouÉ, A.; Blaimont, P.; Chauveau, V.; Miles, D.B.; Guillon, M.; Richard, M.; Badiane, A.; Meylan, S.; et al. Interaction of Hydric and Thermal Conditions Drive Geographic Variation in Thermoregulation in a Widespread Lizard. *Ecol. Monogr.* **2021**, *91*, e01440. https://doi.org/10.1002/ecm.1440.
38. IUCN/SSC. *Guidelines for Reintroductions and Other Conservation Translocations*, version 1.0; IUCN Species Survival Commission: Gland, Switzerland, 2013; ISBN 978-2-8317-1609-1.
39. Germano, J.M.; Bishop, P.J. Suitability of Amphibians and Reptiles for Translocation. *Conserv. Biol.* **2009**, *23*, 7–15. https://doi.org/10.1111/j.1523-1739.2008.01123.x.
40. Besson, A.A.; Cree, A. Integrating Physiology into Conservation: An Approach to Help Guide Translocations of a Rare Reptile in a Warming Environment: Thermal Biology and Conservation of Tuatara. *Anim. Conserv.* **2011**, *14*, 28–37. https://doi.org/10.1111/j.1469-1795.2010.00386.x.
41. Chauvenet, A.L.M.; Ewen, J.G.; Armstrong, D.P.; Blackburn, T.M.; Pettorelli, N. Maximizing the Success of Assisted Colonizations: Maximizing the Success of Assisted Colonization. *Anim. Conserv.* **2013**, *16*, 161–169. https://doi.org/10.1111/j.1469-1795.2012.00589.x.
42. Batson, W.G.; Gordon, I.J.; Fletcher, D.B.; Manning, A.D. REVIEW: Translocation Tactics: A Framework to Support the IUCN Guidelines for Wildlife Translocations and Improve the Quality of Applied Methods. *J. Appl. Ecol.* **2015**, *52*, 1598–1607. https://doi.org/10.1111/1365-2664.12498.
43. Besson, A.A.; Cree, A. A Cold-Adapted Reptile Becomes a More Effective Thermoregulator in a Thermally Challenging Environment. *Oecologia* **2010**, *163*, 571–581. https://doi.org/10.1007/s00442-010-1571-y.
44. Jetz, W.; Ashton, K.G.; La Sorte, F.A. Phenotypic Population Divergence in Terrestrial Vertebrates at Macro Scales. *Ecol. Lett.* **2009**, *12*, 1137–1146. https://doi.org/10.1111/j.1461-0248.2009.01369.x.
45. Ebrahimi, M.; Ebrahimie, E.; Bull, C.M. Minimizing the Cost of Translocation Failure with Decision-Tree Models That Predict Species' Behavioral Response in Translocation Sites: Species Behavior and Decision-Tree Models. *Conserv. Biol.* **2015**, *29*, 1208–1216. https://doi.org/10.1111/cobi.12479.
46. Rummel, L.; Martínez–Abraín, A.; Mayol, J.; Ruiz-Olmo, J.; Mañas, F.; Jiménez, J.; Gómez, J.A.; Oro, D. Use of Wild–Caught Individuals as a Key Factor for Success in Vertebrate Translocations. *Anim. Biodiv. Conserv.* **2016**, *39*, 207–219. https://doi.org/10.32800/abc.2016.39.0207.
47. Caldwell, A.J.; While, G.M.; Wapstra, E. Plasticity of Thermoregulatory Behaviour in Response to the Thermal Environment by Widespread and Alpine Reptile Species. *Anim. Behav.* **2017**, *132*, 217–227. https://doi.org/10.1016/j.anbehav.2017.07.025.
48. Fordham, D.A.; Watts, M.J.; Delean, S.; Brook, B.W.; Heard, L.M.B.; Bull, C.M. Managed Relocation as an Adaptation Strategy for Mitigating Climate Change Threats to the Persistence of an Endangered Lizard. *Glob. Change Biol.* **2012**, *18*, 2743–2755. https://doi.org/10.1111/j.1365-2486.2012.02742.x.
49. Bulova, S.J. Ecological Correlates of Population and Individual Variation in Antipredator Behavior of Two Species of Desert Lizards. *Copeia* **1994**, *1994*, 980. https://doi.org/10.2307/1446721.
50. Zani, P.A.; Tillman, J.L.; Scoular, K.M. Geographic Variation of Movement and Display Behavior of Side-Blotched Lizards (*Uta stansburiana*) Related to Predation Environment. *J. Herpetol.* **2013**, *47*, 85–92. https://doi.org/10.1670/11-114.

51. Monasterio, C.; Shoo, L.P.; Salvador, A.; Siliceo, I.; Díaz, J.A. Thermal Constraints on Embryonic Development as a Proximate Cause for Elevational Range Limits in Two Mediterranean Lacertid Lizards. *Ecography* **2011**, *34*, 1030–1039. https://doi.org/10.1111/j.1600-0587.2010.06905.x.
52. Ward-Fear, G.; Brown, G.P.; Pearson, D.J.; West, A.; Rollins, L.A.; Shine, R. The Ecological and Life History Correlates of Boldness in Free-Ranging Lizards. *Ecosphere* **2018**, *9*, e02125. https://doi.org/10.1002/ecs2.2125.
53. Ma, L.; Sun, B.; Cao, P.; Li, X.; Du, W. Phenotypic Plasticity May Help Lizards Cope with Increasingly Variable Temperatures. *Oecologia* **2018**, *187*, 37–45. https://doi.org/10.1007/s00442-018-4127-1.
54. Milne, T.; Bull, C.M. Burrow Choice by Individuals of Different Sizes in the Endangered Pygmy Blue Tongue Lizard *Tiliqua adelaidensis*. *Biol. Conserv.* **2000**, *95*, 295–301.
55. Souter, N.J.; Bull, C.M.; Lethbridge, M.R.; Hutchinson, M.N. Habitat Requirements of the Endangered Pygmy Bluetongue Lizard, *Tiliqua adelaidensis*. *Biol. Conserv.* **2007**, *135*, 33–45. https://doi.org/10.1016/j.biocon.2006.09.014.
56. Pettigrew, M.; Bull, C.M. The Impact of Heavy Grazing on Burrow Choice in the Pygmy Bluetongue Lizard, *Tiliqua adelaidensis*. *Wildl. Res.* **2011**, *38*, 299. https://doi.org/10.1071/WR11052.
57. Clayton, J.; Bull, C.M. The Impact of Sheep Grazing on Burrows for Pygmy Bluetongue Lizards and on Burrow Digging Spiders: Grazing Impact on Spider Burrow Dynamics. *J. Zool.* **2015**, *297*, 44–53. https://doi.org/10.1111/jzo.12247.
58. Cooper, W. Pursuit Deterrence, Predation Risk, and Escape in the Lizard *Callisaurus draconoides*. *Behav. Ecol. Sociobiol.* **2011**, *65*, 1833. https://doi.org/10.1007/s00265-011-1191-5.
59. Maxim Integrated. DS1923 iButton Hygrochron Temperature/Humidity Logger with 8KB Data-Log Memory | Maxim Integrated. Available online: https://www.maximintegrated.com/en/products/ibutton-one-wire/data-loggers/DS1923.html (accessed on 3 November 2021).
60. R Core Team. *R: A Language and Environment for Statistical Computing*; R Foundation for Statistical Computing: Vienna, Austria, 2021.
61. Muff, S.; Nilsen, E.B.; O'Hara, R.B.; Nater, C.R. Rewriting Results Sections in the Language of Evidence. *Trends Ecol. Evol.* **2022**, *37*, 203–210. https://doi.org/10.1016/j.tree.2021.10.009.
62. Muff, S.; Nilsen, E.B.; O'Hara, R.B.; Nater, C.R. Response to 'Why P Values Are Not Measures of Evidence' by D. Lakens. *Trends Ecol. Evol.* **2022**, *37*, 291–292. https://doi.org/10.1016/j.tree.2022.01.001.
63. Grimm-Seyfarth, A.; Mihoub, J.; Gruber, B.; Henle, K. Some like It Hot: From Individual to Population Responses of an Arboreal Arid-zone Gecko to Local and Distant Climate. *Ecol. Monogr.* **2018**, *88*, 336–352. https://doi.org/10.1002/ecm.1301.
64. Doody, J.S.; McGlashan, J.; Fryer, H.; Coleman, L.; James, H.; Soennichsen, K.; Rhind, D.; Clulow, S. Plasticity in Nest Site Choice Behavior in Response to Hydric Conditions in a Reptile. *Sci. Rep.* **2020**, *10*, 16048. https://doi.org/10.1038/s41598-020-73080-6.
65. Mi, C.; Ma, L.; Wang, Y.; Wu, D.; Du, W.; Sun, B. Temperate and Tropical Lizards Are Vulnerable to Climate Warming Due to Increased Water Loss and Heat Stress. *Proc. R. Soc. B.* **2022**, *289*, 20221074. https://doi.org/10.1098/rspb.2022.1074.
66. Pintor, A.F.V.; Schwarzkopf, L.; Krockenberger, A.K. Extensive Acclimation in Ectotherms Conceals Interspecific Variation in Thermal Tolerance Limits. *PLoS ONE* **2016**, *11*, e0150408. https://doi.org/10.1371/journal.pone.0150408.
67. Rozen-Rechels, D.; Dupoué, A.; Lourdais, O.; Chamaillé-Jammes, S.; Meylan, S.; Clobert, J.; Le Galliard, J. When Water Interacts with Temperature: Ecological and Evolutionary Implications of Thermo-hydroregulation in Terrestrial Ectotherms. *Ecol. Evol.* **2019**, *9*, 10029–10043. https://doi.org/10.1002/ece3.5440.
68. Gilchrist, G.W. Specialists and Generalists in Changing Environments. I. Fitness Landscapes of Thermal Sensitivity. *Am. Nat.* **1995**, *146*, 252–270. https://doi.org/10.1086/285797.
69. Cruz, F.B.; Fitzgerald, L.A.; Espinoza, R.E.; Schulte Ii, J.A. The Importance of Phylogenetic Scale in Tests of Bergmann's and Rapoport's Rules: Lessons from a Clade of South American Lizards: Bergmann's and Rapoport's Rules in Lizards. *J. Evol. Biol.* **2005**, *18*, 1559–1574. https://doi.org/10.1111/j.1420-9101.2005.00936.x.
70. Treilibs, C.E.; Pavey, C.R.; Raghu, S.; M. Bull, C. Weather Correlates of Temporal Activity Patterns in a Desert Lizard: Insights for Designing More Effective Surveys. *J. Zool.* **2016**, *300*, 281–290. https://doi.org/10.1111/jzo.12381.
71. Kearney, M.; Porter, W.P. Mapping the Fundamental Niche: Physiology, Climate, and the Distribution of a Nocturnal Lizard. *Ecology* **2004**, *85*, 3119–3131. https://doi.org/10.1890/03-0820.
72. Paget, S.; Gleiss, A.C.; Kuchling, G.; Mitchell, N.J. Activity of a Freshwater Turtle Varies across a Latitudinal Gradient: Implications for the Success of Assisted Colonisation. *Funct. Ecol.* **2023**, *37*, 1897–1909. https://doi.org/10.1111/1365-2435.14338.
73. Gienapp, P.; Teplitsky, C.; Alho, J.S.; Mills, J.A.; Merilä, J. Climate Change and Evolution: Disentangling Environmental and Genetic Responses. *Mol. Ecol.* **2008**, *17*, 167–178. https://doi.org/10.1111/j.1365-294X.2007.03413.x.

74. Al-Sadoon, K.; Spellerberg, F. Comparison of Thermal Acclimation Effects on the Metabolism of *Chalcides ocellatus* (Desert Lizard) and *Lacerta vivipara* (Cool-Temperate Lizard). *Comp. Biochem. Physiol. Part A Mol. Integr. Physiol.* **1985**, *81*, 939–943.
75. Araújo, M.B.; Ferri-Yáñez, F.; Bozinovic, F.; Marquet, P.A.; Valladares, F.; Chown, S.L. Heat Freezes Niche Evolution. *Ecol. Lett.* **2013**, *16*, 1206–1219. https://doi.org/10.1111/ele.12155.
76. Herrando-Pérez, S.; Ferri-Yáñez, F.; Monasterio, C.; Beukema, W.; Gomes, V.; Belliure, J.; Chown, S.L.; Vieites, D.R.; Araújo, M.B. Intraspecific Variation in Lizard Heat Tolerance Alters Estimates of Climate Impact. *J. Anim. Ecol.* **2019**, *88*, 247–257. https://doi.org/10.1111/1365-2656.12914.
77. Sinervo, B.; Mendez-de-la-Cruz, F.; Miles, D.B.; Heulin, B.; Bastiaans, E.; Villagran-Santa Cruz, M.; Lara-Resendiz, R.; Martinez-Mendez, N.; Calderon-Espinosa, M.L.; Meza-Lazaro, R.N.; et al. Erosion of Lizard Diversity by Climate Change and Altered Thermal Niches. *Science* **2010**, *328*, 894–899. https://doi.org/10.1126/science.1184695.
78. Huey, R.B.; Kearney, M.R.; Krockenberger, A.; Holtum, J.A.M.; Jess, M.; Williams, S.E. Predicting Organismal Vulnerability to Climate Warming: Roles of Behaviour, Physiology and Adaptation. *Philos. Trans. R. Soc. B* **2012**, *367*, 1665–1679. https://doi.org/10.1098/rstb.2012.0005.
79. Muñoz, M.M.; Langham, G.M.; Brandley, M.C.; Rosauer, D.F.; Williams, S.E.; Moritz, C. Basking Behavior Predicts the Evolution of Heat Tolerance in Australian Rainforest Lizards: Physiological Evolution in Australian Skinks. *Evolution* **2016**, *70*, 2537–2549. https://doi.org/10.1111/evo.13064.
80. Campbell-Staton, S.C.; Edwards, S.V.; Losos, J.B. Climate-Mediated Adaptation after Mainland Colonization of an Ancestrally Subtropical Island Lizard, *Anolis carolinensis*. *J. Evol. Biol.* **2016**, *29*, 2168–2180. https://doi.org/10.1111/jeb.12935.
81. Senior, A.F.; Atkins, Z.S.; Clemann, N.; Gardner, M.G.; Schroder, M.; While, G.M.; Wong, B.B.M.; Chapple, D.G. Variation in Thermal Biology of Three Closely Related Lizard Species along an Elevation Gradient. *Biol. J. Linn. Soc.* **2019**, *127*, 278–291. https://doi.org/10.1093/biolinnean/blz046.
82. Sinervo, B. Evolution of Thermal Physiology and Growth Rate between Populations of the Western Fence Lizard (*Sceloporus occidentalis*). *Oecologia* **1990**, *83*, 228–237. https://doi.org/10.1007/BF00317757.
83. Zhang, D.-J.; Tang, X.-L.; Yue, F.; Chen, Z.; Li, R.-D.; Chen, Q. Effect of Gestation Temperature on Sexual and Morphological Phenotypes of Offspring in a Viviparous Lizard, *Eremias multiocellata*. *J. Therm. Biol.* **2010**, *35*, 129–133. https://doi.org/10.1016/j.jtherbio.2010.01.003.
84. Tang, X.-L.; Yue, F.; Yan, X.-F.; Zhang, D.-J.; Xin, Y.; Wang, C.; Chen, Q. Effects of Gestation Temperature on Offspring Sex and Maternal Reproduction in a Viviparous Lizard (*Eremias multiocellata*) Living at High Altitude. *J. Therm. Biol.* **2012**, *37*, 438–444. https://doi.org/10.1016/j.jtherbio.2012.03.002.
85. Roitberg, E.S.; Eplanova, G.V.; Kotenko, T.I.; Amat, F.; Carretero, M.A.; Kuranova, V.N.; Bulakhova, N.A.; Zinenko, O.I.; Yakovlev, V.A. Geographic Variation of Life-History Traits in the Sand Lizard, *Lacerta Agilis*: Testing Darwin's Fecundity-Advantage Hypothesis. *J. Evol. Biol.* **2015**, *28*, 613–629. https://doi.org/10.1111/jeb.12594.
86. While, G.M.; Williamson, J.; Prescott, G.; Horváthová, T.; Fresnillo, B.; Beeton, N.J.; Halliwell, B.; Michaelides, S.; Uller, T. Adaptive Responses to Cool Climate Promotes Persistence of a Non-Native Lizard. *Proc. R. Soc. B* **2015**, *282*, 20142638. https://doi.org/10.1098/rspb.2014.2638.
87. Shine, R.; Wapstra, E.; Olsson, M. Seasonal Shifts along the Oviparity-Viviparity Continuum in a Cold-Climate Lizard Population. *J. Evol. Biol.* **2018**, *31*, 4–13. https://doi.org/10.1111/jeb.13202.
88. Smith, G.D.; Zani, P.A.; French, S.S. Life-history Differences across Latitude in Common Side-blotched Lizards (*Uta stansburiana*). *Ecol. Evol.* **2019**, *9*, 5743–5751. https://doi.org/10.1002/ece3.5157.
89. Llewelyn, J.; Macdonald, S.L.; Moritz, C.; Martins, F.; Hatcher, A.; Phillips, B.L. Adjusting to Climate: Acclimation, Adaptation and Developmental Plasticity in Physiological Traits of a Tropical Rainforest Lizard. *Integr. Zool.* **2018**, *13*, 411–427. https://doi.org/10.1111/1749-4877.12309.
90. Cadby, C.D.; Jones, S.M.; Wapstra, E. Geographical Differences in Maternal Basking Behaviour and Offspring Growth Rate in a Climatically Widespread Viviparous Reptile. *J. Exp. Biol.* **2014**, *217*, 1175–1179. https://doi.org/10.1242/jeb.089953.
91. Niewiarowski, P.H.; Roosenburg, W. Reciprocal Transplant Reveals Sources of Variation in Growth Rates of the Lizard *Sceloporus undulatus*. *Ecology* **1993**, *74*, 1992–2002. https://doi.org/10.2307/1940842.
92. McDonald, S.; Schwanz, L.E. Thermal Parental Effects on Offspring Behaviour and Their Fitness Consequences. *Anim. Behav.* **2018**, *135*, 45–55. https://doi.org/10.1016/j.anbehav.2017.11.007.
93. Frankham, R. Genetic Rescue Benefits Persist to at Least the F3 Generation, Based on a Meta-Analysis. *Biol. Conserv.* **2016**, *195*, 33–36. https://doi.org/10.1016/j.biocon.2015.12.038.

94. Paranjpe, D.A.; Bastiaans, E.; Patten, A.; Cooper, R.D.; Sinervo, B. Evidence of Maternal Effects on Temperature Preference in Side-Blotched Lizards: Implications for Evolutionary Response to Climate Change. *Ecol. Evol.* **2013**, *3*, 1977–1991. https://doi.org/10.1002/ece3.614.
95. Artacho, P.; Saravia, J.; Perret, S.; Bartheld, J.L.; Le Galliard, J.-F. Geographic Variation and Acclimation Effects on Thermoregulation Behavior in the Widespread Lizard *Liolaemus pictus*. *J. Therm. Biol.* **2017**, *63*, 78–87. https://doi.org/10.1016/j.jtherbio.2016.11.001.
96. Shine, R.; Brown, G.P. Adapting to the Unpredictable: Reproductive Biology of Vertebrates in the Australian Wet–Dry Tropics. *Philos. Trans. R. Soc. B* **2008**, *363*, 363–373. https://doi.org/10.1098/rstb.2007.2144.
97. Schlesinger, C.A.; Christian, K.A.; James, C.D.; Morton, S.R. Seven Lizard Species and a Blind Snake: Activity, Body Condition and Growth of Desert Herpetofauna in Relation to Rainfall. *Aust. J. Zool.* **2010**, *58*, 273. https://doi.org/10.1071/ZO10058.
98. Treilibs, C.E.; Pavey, C.R.; Gardner, M.G.; Ansari, M.H.; Bull, C.M. Spatial Dynamics and Burrow Occupancy in a Desert Lizard Floodplain Specialist, *Liopholis slateri*. *J. Arid Environ.* **2019**, *167*, 8–17. https://doi.org/10.1016/j.jaridenv.2019.04.004.
99. Ritchie, E.G.; Bradshaw, C.J.A.; Dickman, C.R.; Hobbs, R.; Johnson, C.N.; Johnston, E.L.; Laurance, W.F.; Lindenmayer, D.; McCarthy, M.A.; Nimmo, D.G.; et al. Continental-Scale Governance and the Hastening of Loss of Australia's Biodiversity: Editorial. *Conserv. Biol.* **2013**, *27*, 1133–1135. https://doi.org/10.1111/cobi.12189.
100. Geyle, H.M.; Tingley, R.; Amey, A.P.; Cogger, H.; Couper, P.J.; Cowan, M.; Craig, M.D.; Doughty, P.; Driscoll, D.A.; Ellis, R.J.; et al. Reptiles on the Brink: Identifying the Australian Terrestrial Snake and Lizard Species Most at Risk of Extinction. *Pac. Conserv. Biol.* **2020**, *27*, 3–12. https://doi.org/10.1071/PC20033.
101. Germano, J.; Ewen, J.G.; Mushinsky, H.; McCoy, E.; Ortiz-Catedral, L. Moving towards Greater Success in Translocations: Recent Advances from the Herpetofauna: Greater Success in Translocations. *Anim. Conserv.* **2014**, *17*, 1–3. https://doi.org/10.1111/acv.12172.

Disclaimer/Publisher's Note: The statements, opinions and data contained in all publications are solely those of the individual author(s) and contributor(s) and not of MDPI and/or the editor(s). MDPI and/or the editor(s) disclaim responsibility for any injury to people or property resulting from any ideas, methods, instructions or products referred to in the content.
